# Towards an agenda for implementation science in global health: there is nothing more practical than good (social science) theories

**DOI:** 10.1136/bmjgh-2016-000181

**Published:** 2017-03-24

**Authors:** Sara Van Belle, Remco van de Pas, Bruno Marchal

**Affiliations:** 1 Health Policy Unit, Department of Public Health, Institute of Tropical Medicine, Antwerp, Belgium; 2 Health Services Organisation Unit, Department of Public Health, Institute of Tropical Medicine, Antwerp, Belgium

**Summary box**

- There have been calls for more use of theory in implementation studies and implementation science.
- There is currently little attention for systematically using theories from social sciences in implementation science.
- Realist evaluation, and other theory-driven evaluation and research approaches, provide a useful approach to better build implementation science studies on theories as well as to test and develop theories.

Ridde recently called for a commitment to implementation science and for ‘better quality research in order to have a greater understanding of how to implement health interventions’.^1^ He argued for better use of theory and implementation science studies that contribute to theory. This is indeed of paramount importance, especially for interventions in complex settings such as health systems. In practice, however, few researchers take up the challenge; all too often, implementation studies adopt a mixed methods approach that is a-theoretical.

If there is a lack of theory in the field of implementation science, it is not for want of theories. In the case of health governance, for instance, theory development and empirical research testing such theories have been taking place in the fields of economics and political science. However, there has been little, if any, systematic crossing over of recent governance theories to the field of health policy and systems research. This does not only apply to health (system) governance; research on health worker motivation and strategic decision-making at operational level, for instance, could use a healthy injection of recent theories and methods from other disciplines.

In applied research fields, such as global environmental studies, there are more signs of effective cross-pollination. The study of global climate change is an example of how the integration of different paradigms yields new theoretical insights that drive empirical research; for example, the late Elinor Ostrom's conceptualisation of polycentricity in efforts to address global environmental change.^2^ The linkage of ‘social’ and ‘ecological’ systems furthers the understanding of actual policy implementation problems that are of a social nature in the field of environmental science (see the journals *Global Environmental Change*, *Global Environmental Politics and Global Challenges* for other examples). In the field of global health, we are happy to note that *BMJ Global Health* offers a new avenue for such transdisciplinary research.

Using existing social science theories to *inform* implementation is important as it helps to not reinvent the wheel; using theory to inform *research* in implementation matters too. In order to effectively learn from the implementation of health policies, programmes or interventions, existing theory can help inform study design in (at least) two ways. First, not only is it important to demonstrate the effectiveness of a policy, programme or intervention and to understand their implementation process, the causal processes that underlie the intervention and the deeper societal change they envisage also need to be understood. Theories (in the sense of condensed and tested knowledge) can provide hypotheses about causal processes and therefore allow researchers to explicitly test whether they hold or not. However, all too often, we see implementation studies that do not explicitly and systematically look for causal processes. Second, theories may offer clues regarding the contextual conditions in which a policy, programme or intervention is likely to work (or not). Needless to say, this can inform study design so as to enable the testing of these propositions and potentially improve claims of external validity.

One step beyond theory-informed implementation science is theory-building implementation science. We agree again with Ridde and would like to specify that implementation science is well placed to contribute to building of theories of the middle range. Merton defined these as “theories that lie between the minor but necessary working hypotheses (…) and the all-inclusive systematic efforts to develop a unified theory that will explain all the observed uniformities of social behavior, social organization and social change.”^3^ While there are arguably no unified theories in social science, examples of grand theories that attempt to explain core social issues include functionalism, symbolic interactionism and social exchange theory. Examples of theories of the middle range are cognitive dissonance, self-fulfilling prophecy, reference group theory, etc. Such empirically testable theories have the potential not only to inform implementation science studies: they can be refined through repeated studies, gradually gaining in validity and thus in capacity to guide the implementation. In this way, nothing is more practical than a good theory.^4^

One approach to research that is explicitly built on developing middle range theories is realist evaluation (or *realist research*). Developed by Pawson and Tilley in the late 1990s^5^ realist evaluation is inspired by critical realism, of which Ridde says that “theoretical approach of critical realism, (which) is not well-tested in LMICs [low- and middle-income countries]”.^1^ This should not be surprising: critical realism is a philosophy of science, developed by Bhaskar, Archer *et al*.^6^ Realist research adopted some key principles of critical realism^7^ and is much more used in health policy and systems research than critical realism itself. The clarion call of realist research ‘to find out not only what works, but how, for whom and why?’ is nowadays widely echoed in this field. In our experience, this is with just cause: realist research (and theory-driven approaches in general) is well suited to demonstrate the interplay between policy, programme or intervention, context, actors, causal mechanisms and outcomes. It is, indeed, complexity-sensitive.^8^ Realist research explicitly aims at building and refining middle range theory through the analytical comparison between cases and by using the configuration of contextual conditions, mechanisms and outcomes as a heuristic. The gradual development of theory during analysis provides a bridge between cases.

These days, realist research seems to be fashionable in health policy and system research. In our view, one reason is it provides health researchers from biomedical backgrounds with a pragmatic handle to understand the nuts and bolts of interventions, programmes and policies, and showing in the process how social science theory is actually built and applied. It stimulates the mobilisation of ideas and theories from other disciplines—indeed, it even requires it—and this helps in analysing of the complex problems that health policy and systems researchers, as well as implementation scientists, look into.

We take the example of research of health policy implementation. In our view, such studies in essence need to cover the upstream processes of policy formulation and development, their translation into programmes, the required conditions (‘enabling environment’) needed to achieve successful implementation and the feedback loops from the operational level back to policy formulation. They need to take into account actors, positions, power, interests and relations. Theories from political science, economics, public administration, organisational theory and organisational psychology (to name just a few relevant disciplines) can inform the research of each stage, interface or feedback loop. Moreover, these fields also provide a number of theories on policy implementation. Realist researchers explore which theories may be used to develop an initial middle range theory. They also elicit the assumptions of the policymakers, programme managers, implementing providers, citizens, etc who all have ideas about why the policy will be successfully implemented or not. The end result is an initial hypothesis (middle range theory) that will guide their further study into the effectiveness of the policy in question, why it is successfully implemented (or not), who is implementing (or not), how and under which conditions.

The down-side of the current popularity of realist research is that researchers increasingly use the slogan and label their studies as ‘realist’ without applying its principles.^9^ ^10^ This reflects a similar development in mixed methods studies, or multiple case studies, which too are often developed a-theoretically. However, we also acknowledge that the application of realist research is by no means easy, especially for beginning researchers with a scant background in social sciences and in theory development. Developing good theories from implemented health interventions indeed may require the mobilisation of multiple social science theories and concepts. Social science researchers can help identify good starter theories, and tease out the mechanisms at work, from the level of the cognitive and relational drivers of behaviour up to organisational and wider institutional mechanisms.

Testing theories has its own challenges. We take here again the example of health governance research. Global health research has made some forays in policy analysis, using older, yet still powerful, frameworks. Much less has it engaged with current governance theories, which reflect an actor-oriented, institutionalist turn in political science and economics.^11^ Such theories are not easy to operationalise in terms of interventions, and testing them in the (health) settings in low-income and middle-income countries (LMICs) is not evident. Furthermore, almost all governance theories originate in high-income countries—even theories of global governance. As Ridde asserts in relation to policy analysis, these could possibly be adapted to the situation of LMICs, and fragile settings. However, in practice, a singular perspective, principal-agent theory looms large over current health governance research^12^ ^13^ perhaps unsurprisingly: as with any research field, the popularity of theories in global health research is subject to paradigmatic power and politics.^14^ At this moment, critical theories from political science, cognisant of the networked global society and its power dynamics (see for instance^2^ ^15–17^) hold noticeably less sway in this field. Here, the ‘critical’ in critical realism creeps in through the back door. Equity-focused implementation science needs to speak truth to power; it needs to be able to develop critiques of society's structural conditions and expose the role of actors, agency, politics, power relations and discourse (see Storeng^18^ for a nice example) and adopt theoretical approaches that allow exactly that.

To conclude, the future of implementation science is promising. We would call on interdisciplinary and social science researchers to engage in the field, and to also facilitate the use of social science theories and methods in implementation science in global health. We must not leave the field with ‘a chaos of methods (and theories) in largely unproductive competition’.^14^ Now we have the opportunity to produce better implementation science through theory.
